# C-Terminal Domain Deletion Enhances the Protective Activity of *cpa/cpb* Loaded Solid Lipid Nanoparticles against *Leishmania major* in BALB/c Mice

**DOI:** 10.1371/journal.pntd.0001236

**Published:** 2011-07-12

**Authors:** Delaram Doroud, Farnaz Zahedifard, Alireza Vatanara, Yasaman Taslimi, Rouholah Vahabpour, Fatemeh Torkashvand, Behrooz Vaziri, Abdolhossein Rouholamini Najafabadi, Sima Rafati

**Affiliations:** 1 Molecular Immunology and Vaccine Research Laboratory, Pasteur Institute of Iran, Tehran, Iran; 2 Department of Pharmaceutics, School of Pharmacy, Tehran University of Medical Sciences, Tehran, Iran; 3 Hepatitis and AIDS Department, Pasteur Institute of Iran, Tehran, Iran; 4 Biotechnology Research Center, Pasteur Institute of Iran, Tehran, Iran; Institut Pasteur, France

## Abstract

**Background:**

We have demonstrated that vaccination with pDNA encoding cysteine proteinase Type II (CPA) and Type I (CPB) with its unusual C-terminal extension (CTE) can partially protect BALB/c mice against cutaneous leishmanial infection. Unfortunately, this protection is insufficient to completely control infection without booster injection. Furthermore, in developing vaccines for leishmaniasis, it is necessary to consider a proper adjuvant and/or delivery system to promote an antigen specific immune response. Solid lipid nanoparticles have found their way in drug delivery system development against intracellular infections and cancer, but not *Leishmania* DNA vaccination. Therefore, undefined effect of cationic solid lipid nanoparticles (cSLN) as an adjuvant in enhancing the immune response toward leishmanial antigens led us to refocus our vaccine development projects.

**Methodology/Principal Findings:**

Three pDNAs encoding *L. major* cysteine proteinase type I and II (with or without CTE) were formulated by cSLN. BALB/c mice were immunized twice by 3-week interval, with cSLN-pcDNA-*cpa/b*, pcDNA-*cpa/b*, cSLN-pcDNA-*cpa/b^-CTE^*, pcDNA-*cpa/b^-CTE^*, cSLN, cSLN-pcDNA and PBS. Mice vaccinated with cSLN-pcDNA-*cpa/b^-CTE^* showed significantly higher levels of parasite inhibition related to protection with specific Th1 immune response development, compared to other groups. Parasite inhibition was determined by different techniques currently available in exploration vacciation efficacy, i.e., flowcytometry on footpad and lymph node, footpad caliper based measurements and imaging as well as lymph node microtitration assay. Among these techniques, lymph node flowcytometry was found to be the most rapid, sensitive and easily reproducible method for discrimination between the efficacy of vaccination strategies.

**Conclusions/Significance:**

This report demonstrates cSLN's ability to boost immune response magnitude of *cpa/cpb^-CTE^* cocktail vaccination against leishmaniasis so that the average parasite inhibition percent could be increased significantly. Hence, cSLNs can be considered as suitable adjuvant and/or delivery systems for designing third generation cocktail vaccines.

## Author Summary

Cutaneous leishmaniasis (CL) is the most common form of leishmaniasis with an annual incidence of approximately 2 million cases and is endemic in 88 countries, including Iran. CL's continued spread, along with rather ineffectual treatments and drug-resistant variants emergence has increased the need for advanced preventive strategies. We studied Type II cysteine proteinase (CPA) and Type I (CPB) with its C-terminal extension (CTE) as cocktail DNA vaccine against murine and canine leishmaniasis. However, adjuvants' success in enhancing immune responses to selected antigens led us to refocus our vaccine development programs. Herein, we discuss cationic solid lipid nanoparticles' (cSLN) ability to improve vaccine-induced protective efficacy against CL and subsequent lesion size and parasite load reduction in BALB/c mice. For this work, we evaluated five different conventional as well as novel parasite detection techniques, i.e., footpad imaging, footpad flowcytometry and lymph node flowcytometry for disease progression assessments. Vaccination with cSLN-*cpa*/*cpb^-CTE^* formulation showed highest parasite inhibition at 3-month post vaccination. Immunized mice showed reduced IL-5 level and significant IFN-ã increase, compared to control groups. We think our study represents a potential future and a major step forward in vaccine development against leishmaniasis.

## Introduction

Leishmaniasis is one of the most important vector borne infections that can cause a spectrum of diseases, ranging from a clinically silent process to a fatal progressive disease in human. It is a major public health crisis in many countries including Iran resulting in an estimated of 12 million new cases occurrence, each year (World Health Organization website, http://www.who.int/vaccine_research/diseases/soa_parasitic/en/index3.html). This parasitic disease is diagnosed as three clinical forms named cutaneous, mucocutaneous and visceral leishmaniasis. Clinical manifestation of the disease depends on both the species involved and the host. The tissue lesion in cutaneous form, can last for months or years before healing. An interesting feature is that despite the disappearance of the lesion and resistance to reinfection, residual parasites remain in the host, probably for a very long time, if not forever [1]. Current curative therapies for cutaneous leishmaniasis are costly, often poorly tolerated and not always effective. This disease is one the few parasitic diseases likely to be controllable with vaccination [2]. Generally; vaccination is largely protein-based and requires direct administration of dead or attenuated parasite, recombinant proteins, or virus-like particles. For targets resembling intracellular pathogens like *Leishmania* species, such vaccines generate incomplete immune responses and fail to induce protective affects as usually generate only antibody-mediated (humoral) immune responses and often require periodic booster injections [2], [3]. However, cell-mediated immune responses are required for clearance of such parasite and generation of cytotoxic T-lymphocyte (CTL) cells that kill infected cells. Currently, only attenuated live organism vaccines generate significant cell-mediated immune responses, but these are associated with certain safety concerns and can be difficult to manufacture consistently [2], [3]. DNA vaccination offers an attractive alternative to traditional non replicating vaccine strategies. Intracellular production of antigens from delivered DNAs can result in both humoral and cellular immune responses. DNA-based vaccines also offer practical advantages as well, mostly because of the capability of developing countries to cheaply and rapidly produce pDNA from bacteria. Furthermore, it is possible to formulate several antigens from different stages of the parasite life cycle or different subspecies as one shot of vaccine [4]. However, despite decades of research, safe and efficient delivery of pDNA to initiate proper immune responses remains one of the major drawbacks in bringing DNA vaccination into clinical trials. Synthetic particle carrier systems are known as one of the important tools for improvement of current performed DNA vaccines. In such approaches, pDNA is encapsulated into, or complexed via electrostatic interaction with a synthetic carrier, resulting in fabrication of particles with a size ranging from nanometers (nm) to few micrometers. The pDNA release from these particles has proven to be a very effective delivery strategy as the passive targeting to antigen presenting cells (APCs) by size exclusion mechanism, protection from nuclease degradation, cellular uptake enhancement, antigen depot formation at the injection site and controlling the release rates of pDNA that might be important for timing immune responses, are all likely to be occurred [5].

Lipid-based delivery systems represent one of the most advanced drug delivery technologies to date. Different lipid-based adjuvants are introduced and evaluated for pDNA formulation, e.g. Solid lipid nanoparticles (SLN) [based on class I of lipids], liposomes, Transfersomes, niosomes and virosomes [based on class II of lipids] and Micelles, emulsions [based on class III of lipids] [6]. In general, the common ground of these systems for transfection is the need for cationic lipids such as 1, 2-dioleoyl-3-trimethylammonium (DOTAP) to facilitate pDNA binding. A neutral helper lipid such as L-alpha-dioleoyl phosphatidylethanolamine (DOPE) or cholesterol is also required to increase the transfection properties of the pDNA/lipid complexes. Several authors have administered liposomes, niosomes and other lipid based systems for pDNA delivery [6]. But despite offering a number of technological advantages over other existing transfection reagents, SLN utility for DNA vaccination has not been conveniently investigated *in vivo*. As SLN can be manufactured in large scale and under favorable technological parameters without the need for organic solvents and have an acceptable stability that facilitates their manipulation for different processes such as lyophilization and steam sterilization [7], , they may become a potentially valuable addition and promising alternative to the well-established dossier of non-viral transfection agents leading by cationic liposomes.

*Leishmania* express large quantities of cysteine proteinases (CP) which are members of the papain superfamily [9]. In *Leishmania major* (*L. major*), two most important CPs have been described. CPA is a type II cysteine proteinase which is expressed at higher level in amastigote stage and stationary phase promastigote. CPB is a type I cysteine proteinase which present maximally at the amastigote developmental stage and is encoded with an unusual C-terminal extension [10].The presence of this highly variable CTE differentiates Leishmanial rCPB from the other CPs in the papain superfamily [10], [11], [12]. CTE can be glycosylated and partially removed by proteolytic cleavage during processing of the enzyme to its mature form [11]. Hence, CTE fragment is not crucial for enzyme activity and intracellular trafficking, although it is highly immunogenic and responsible for immune evasion and play a role in the diversion of the host immune response [11], [12]. We have reported that antigenic rCTE of *L. infantum* elicitated a predominant IgG2 response in asymptomatic dogs and *in vitro* proliferation of PBMCs. Immunization with CTE also displayed both type 1 and 2 immune signatures in experimental murine model of *L. infantum* infection and therefore is not protective as a vaccine candidate [13].

Furthermore, we had demonstrated that the *cpa/cpb* cocktail is more protective against cutaneous leishmanial infection than the separate forms [14]. Therefore, there was still a need to study the effect of CTE deletion in this cocktail vaccine against *L. major*.

Despite the proven antigenicity and immugenicity of these DNA vaccine candidates, the largest drawback of this kind of vaccination is the obscurity in intracellular delivery of pDNA that causes low levels of gene expression (transfection) which in turn limits the resulting immune responses [15]. Therefore, pDNA must use with a proper and safe formulation in order to coordinate innate and adaptive immune responses and generate strong immunity.

Recently, several immunoadjuvants like BCG, G-CSF and CpG-ODN and also various delivery systems like PLGA microspheres and liposomes have been used to potentiate the immune responses against *Leishmania* antigens [16].

Herein, we used cationic solid-lipid nanoparticle (cSLN) as a non viral transfection agent for delivery of the cocktail DNA vaccine. In our previous studies, cSLN formulation as a delivery system have revealed comparable efficiency/cytotoxicity ratio to linear PEI-25 kDa-pDNAs polyplexes, protected *cpa*, *cpb-^CTE^* and *cpb* genes from extracellular enzymatic degradation and also exhibited considerable low cytotoxicity [17]. In this study, these characterized formulations of cocktail vaccine candidates were evaluated for their immune induction potential in BALB/c mice as sucesptible animal model.

## Materials and Methods

### Materials and chemicals

All solutions were prepared using MilliQ™ ultrapure (Milli-Q-System, Millipore, Molsheim, France) and apyrogenic water to avoid surface-active impurities. Cetyl palmitate, tween 80 and cholesterol were purchased from Merck (Darmstadt, Germany). *N*-[1-(2,3-Dioleoyloxy) propyl]-*N*,*N*,*N* trimethylammonium chloride (DOTAP), Sodium dodecyl sulfate (SDS) were purchased from Sigma–Aldrich (Deisenhofen, Germany) The materials applied for PCR, enzymatic digestion and agarose gel electrophoresis were acquired from Roche Applied Sciences (Mannheim, Germany). Cell culture reagents including Fetal Calf Sera (FCS), M199 medium, HEPES, L-glutamine, adenosine, hemin, gentamicin, and RPMI were sourced from GIBCO (Gibco, Life Technologies GmbH, Karlsruhe, Germany) and Sigma (Germany) respectively.

### cSolid lipid nanoparticles preparation

The cSLN suspension was produced by a technique previously described by Doroud *et al*. [17]. Briefly, desired amount of DOTAP (0.4% w/v) was dissolved in hot aqueous phase which was then added to the melted cetyl palmitate and cholesterol (5.1% w/v) phase containing tween 80 as a nonionic surfactant at 3.2:1 molar ratio. Emulsification was carried out by stirring the mixture at 2000 rpm by a mechanical stirrer (IKA®, Germany) for 10 min at 90°C. Samples were then homogenized using a high shear homogenizer (IKA®, Germany) at 18,000 *g* for 15 min. cSLN dispersion was obtained by direct cooling of hot O/W microemulsion on an ice-bath while stirring at 1000 rpm. cSLNs were washed by centrifugation (6000 *g*, 10 min, three times) using 100 kDa Amicon® Ultra centrifugal filters (Millipore, Schwalbach/Ts, Germany) to purify the suspension from the excess amounts of surfactant. Endotoxin concentration in the cSLN formulation was determined by limulus amoebocyte lysate (LAL) assay (LAL Kit, Charles Riever Endosafe, T2092 CTK7, and USA).The physicochemical stability of the formulations were evaluated at 4±1°C, 25±1°C at dark for 1 month at regular time intervals via observation of any changes in suspension clarity, particle size and zeta potential assessments.

### Plasmid construction and purification

pGEM-*cpa*, pGEM-*cpb* and pGEM*-cpb^-CTE^* were available from our previous studies [17] and each of antigenic fragments were subcloned into pcDNA 3.1(−) vector (Invitrogen). Plasmid DNAs were transformed into the DH5α *E. coli* strain and purified by alkaline lysis method (QIAGEN Endofree Plasmid Giga Kit) and then confirmed by PCR and digestion (data not shown). In all three constructs (pcDNA -*cpa*, pcDNA -*cpb*, and pcDNA –*cp*b^-CTE^), the *cpa*, *cpb*, and *cpb^-CTE^* open reading frames were under control of the CMV promoter, inserted downstream of a Kozak consensus sequence and in frame with an initiation codon. The total concentration and purity of pDNAs were determined by NanoDrop® ND-1000 spectrophotometer (Labtech, UK).

### Expression and purification of recombinant CPA, CPB and CPB^-CTE^ for ELISA and cytokine assays

Construct corresponding to pQE-*cpa* and pQE-*cpb* were produced in fusion form with an *N*-terminal histidine (6XHis-tag) for expression and purification of rCPA and rCPB, as previously described [14]. The *cpb^-CTE^* gene was subcloned into the cloning site of the bacterial expression vector pET-23a expression plasmid, downstream of the T7 promoter. The *E. coli* strain BL21 (DE3) was transformed with pET-*cpb^-CTE^* and grown at 37°C in 100 ml LB medium supplemented with 100 µg/ml ampicillin and 25 µg/ml chloramphenicol.

The culture was induced with 1 mM IPTG at an OD_600_ of 0.8 and grown for a further 4.5 h at 37°C. Cells were centrifuged at 8000 rpm for 20 min. Bacterial pellets were dissolved in lysis buffer [50 mM Tris–HCl (pH = 8), 100 mM NaCl and 1 mM EDTA] and frozen overnight at −20°C. After centrifugation (10,000 *g*, 15 min at 4°C) pellets were washed extensively with washing buffer [20 mM Tris–HCl (pH 8), 20 mM NaCl and 1 mM EDTA]. Inclusion Bodies (IB) were purified by imidazole-SDS-Zn reverse staining method. The purified recombinant protein was concentrated by ultrafiltration using Amicon Filter (MWCO: 10 kDa) and dialysed against PBS. Protein concentration was determined with BCA assay kit (Pierce, Rockford, USA). Purified recombinant proteins were analyzed by SDS-PAGE and Coomassie blue staining to assess the integrity and purity of proteins. These proteins were recognized by previously prepared rabbit anti-CPB antisera using western blot technique [12].

### Complexation between cSLN and plasmid constructs

cSLN–pDNA complexes were prepared by adding volumes corresponded to 650 µg of each purified pDNA (pcDNA-*cpa*, pcDNA-*cpb*, pcDNA-*cpb^-CTE^*) to cSLN suspension at a DOTAP:pDNA ratio of 6∶1 (w/w) and 60 min incubation at room temperature separately, as described before [17]. The final formulations were named as S_p_a, S_p_b and S_p_b^-CTE^ respectively. Complete condensation of pDNAs, complexation with cSLN and the ability of the formulation to protect pDNAs from *DNase* I digestion were analyzed as previously demonstrated by agarose gel electrophoresis [17].

### Characterization of the cSLN-pDNA formulations *in vitro*

#### Measurement of size and zeta potential

The sizes of cSLNs and cSLN–pDNA complexes were determined by photon correlation spectroscopy (PCS). For this purpose, the SLN in water dispersions were diluted in the ratio of 1∶100 before analysis. Both size and zeta potential measurements were performed on a Malvern Zetasizer 3000 (Malvern Instruments, Worcestershire, UK). The analysis was performed on each sample immediately after preparation of the cSLNS and 2 hrs after preparing the cSLN-pDNA complex in milli Q water.

#### Gel retardation analysis

Gel retardation assay was applied to determine complex formation between pDNA (5 µg) and cSLNs (DOTAP different weight ratios). For this purpose, 15 µl aliquots of the complexes samples (containing 0.375 µg of pDNA) were subjected to electrophoresis on a 0.8% agarose gel containing 0.5 µg/µl ethidium bromide after mixing with 7 µl loading buffer (0.25% bromophenol blue and 30% glycerol). Electrophoresis was carried out in 80 V for about 2 h in 1X TBE running buffer. The bands were observed with a transilluminator (Bio Doc-ITTM System, UK). Images were captured using a digital camera.

#### *DNase* I protection assay

In order to assess protection ability of cSLNs for loaded pDNAs, *DNase* I was added to cSLN–pDNA complexes to a final concentration of 0.8 U *DNase* I to 5 µg DNA and the mixtures were incubated at 37°C for 1 hr. Then SDS solution was added to the samples to a final concentration of 1% to release DNA from cSLNs. Samples were then analyzed by electrophoresis on agarose gel 0.8% containing ethidium bromide and the integrity of the DNA in each sample was visualized and compared with free DNA as control.

### Vaccination studies

#### Mice, parasites and ethical considerations

The present study was approved by ethical committee at Pasteur Institute of Iran (Education office dated January, 2008). All animal maintenance and blood sample collections were taken under supervision of animal care office of Pasteur Institute of Iran.

Female BALB/c mice, (6–8 weeks old, weighting 20±5 g) were obtained from the Pasteur Institute of Iran and were housed in plastic cages with free access to tap water and standard rodent pellets in an air-conditioned room under a constant 12∶12 h light-dark cycle at room temperature with relative humidity (50–60%).

The enhanced GFP gene (*egfp*) transfected *L. major* strain, (MRHO/IR/75/ER) was kept in a virulent state by continuous passage in BALB/c mice. A homogenized lymph node (LN) from an infected BALB/c mouse was cultured in RPMI-1640 media supplemented with 10% fetal calf serum and 100 µg of gentamycin/ml. Stationary phase promastigotes were harvested by centrifugation at 3000 rpm for 10 min at 4°C. The pellet was washed by PBS (8 mM Na2HPO4, 1.75 mM KH2PO4, 0.25 mM KCl, 137 mM NaCl) and resuspended at a concentration of 3×10^6^ cells/ml. 50 µl of this preparation was injected subcutaneously in the left hind footpad (FP) of each mice.

#### Schedule of animal experiments

Seven groups, each including 13 mice, were immunized in the right hind footpad (FP), using an insulin syringe and a 29-gauge needle (Becton Dickinson, NJ) in a total volume of 40–50 µl. The first group (G1) was immunized with the cocktail of Qiagen® purified pcDNA-*cpa* and pcDNA*-cpb* (50 µg of each plasmid) formulated with cSLN (named as S_p_a/b or cSLN-pcDNA-*cpa/b*). The second group (G2) was immunized with the same amount of non formulated antigens, dissolved in PBS (pcDNA-*cpa/b*). The third group (G3) was immunized with the cocktail of Qiagen® purified pcDNA-*cpa* and pcDNA*-cpb^-CTE^* (50 µg of each plasmid) formulated with cSLN (named as S_p_a/b^-CTE^ or cSLN-pcDNA-*cpa/b^-CTE^*). The forth group (G4) was immunized with the same amount of non formulated pDNAs, dissolved in PBS (pcDNA-*cpa/b^-CTE^*). Control groups received unloaded cSLN formulation (G5), the pcDNA plasmid backbone (100 µg) formulated with cSLN (G6) and PBS (G7). Three weeks later, the same regimens were administered to all the groups, as booster immunization. The vaccinated footpad of the animals was carefully observed for local tolerance assessment at site of injection at 1 and 24 hr after vaccine administration.

Mice were challenged 3 weeks after booster immunization as metioned in the previous section and infection progress was followed as described in the next sections. Blood samples were taken from the eye vein of each individual mouse before and 9 weeks post challenge. The sera were pooled in each group and kept at −20°C untilused. Prior to challenge, 3 mice form each group (n = 13) were sacrificed and their lymph nodes (LNs) were existed and tested for IFN-γ and IL-5 production. At the 9^th^ week post challenge, 3 other mice from each group were euthanized and their LNs were used for parasite burden assesments via flow cytometry and microtitration methods and cytokine profiling. The infected footpads of these mice (n = 3) were also dissected to be evaluated by flow cytometry technique for parasite inhibition assessments. Four mice from each group were randomely selected at this time (9^th^ week post challenge), temporarily anesthetized and imaged for pixel counting and measurement of the lesions asthe detailed procedures are described in the next sections.

### Flow-up of the disease progression

#### A- Footpad assessments. Determination of footpad swelling

FP swelling was weekly monitored by measuring the increase in thickness of the inoculated FP and the uninfected contra lateral FP with a metric caliper.

#### Determination of the footpad parasitic load by flow cytometry

Flow cytometry was used to quantify cells by intracellular fluorescence as an indicator of *Leishmania* infection and replication. Therefore to evaluate the relative efficacy of the vaccination protocols, three mice from each group (n = 10) were euthanized at 9 weeks post challenge and the infected FPs were homogenized with glass homogenizers in 3 ml of PBS under sterile conditions. Normal non infected FP was also removed and processed in the same manner as control. The recovered viable cell suspension was washed with pre-cold PBS (pH 7.2), and monitored using an Epi-fluorescence microscope (Nikon, E200, Japan). Afterward, cells were directly applied to a Partec PASIII flow cytometer (Partec GmbH, Germany) using a bivariate scatter plot of fluorescence versus forward scatter, by gate setting with uninfected FP cells. A minimum of 10,000 events from the viable transfected cell population per sample were analyzed. Parasite inhibition was measured in terms of the decrease in intensity of green fluorescence in the FP of vaccinated animals. This was calculated by comparing the intensity of fluorescence in vaccinated and non vaccinated groups using following formula, (Eq. 1) [18]. Confidence interval with 95% (IC95%) was calculated for each average of parasite inhibition percent considering the lowest and highest values of fluorescent obtained from non vaccinated control groups.
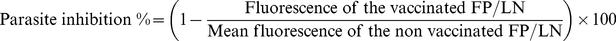
(1)


#### Detection of EGFP-transfected *L. major* in the foodpad of BALB/c mice by imaging system

To demonstrate the *in vivo* level of infection, the infected FPs of four mice per each group (n = 10) were imaged at 9^th^ week after challenge, with the KODAK Image Station 4000 Digital Imaging System. Briefly, all animals were treated with a depilatory substance (Nair) to remove hair from their FPs to reduce background auto fluorescence. Afterward mice were temporarily anesthetized with the mixture of Xylazine 2% (7.5 µl), Ketamine 10% (30 µl) and saline solution (260 µl) per mice intraperitoneally and then imaged. Pixel counting and measurement of the lesions were performed using KODAK molecular image software version 5.3. Measurements were reported as “sum green intensity”, a quantitative measurement defined as the number of green pixels in a given area multiplied by the average intensity of each pixel.

#### B-Parasite burden determination. Conventional microtitration method

Three mice from each group (n = 10) were sacrificed 9 weeks after challenge and parasite burdens were determined as follow: LNs were excised, weighed and then homogenized with a tissue grinder in 2 ml of Schneider's Drosophila medium supplemented with 20% heat-inactivated fetal calf serum and Gentamicin (0.1%). Under sterile conditions serial dilutions ranging from 1 to 10^−20^ were prepared in 96 well micro titration plates. After 7 and 15 days of incubation at 26°C, plates were examined with an inverted microscope at a magnification of 40×. The presence or absence of motile promastigotes was recorded in each well. The final titer was the last dilution for which the well contained at least one parasite [19], [20]. Parasite burden and parasite inhibitions according to this technique were calculated using Eq. (2) and (3). For this purpose, parasite burden for each individual mouse was calculated according to Eq. 2 and the mean values per each group were used for calculation of the parasite inhibition percent according to the Eq. 3. Same as Eq. 1, IC95% was calculated for each vaccinated group, considering lower and higher fluorescence values of the control groups.
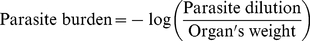
(2)

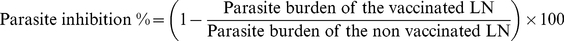
(3)


#### Determination of the lymph node parasite burden directly by flow cytometry

Flow cytometry was used to quantify the lymphatic cells by intracellular fluorescence. Therefore, isolated draining LNs from individual mice (pool of 3 LNs) were homogenized into single cell suspensions using cell dissociation sieves under sterile conditions. The tissue suspensions were then centrifuged at 280×*g* for 5 min at 4°C to precipitate most of the debris and to collect amastigotes in the supernatant. LN of a non infected mouse as control was also removed and processed in the same manner. The GFP-expressing amastigotes of LN cells suspension was monitored using an Epi-fluorescence microscope (Nikon, E200, and Japan). Afterward, cells were directly applied to a Partec PASIII flow cytometer (Partec GmbH, Germany) using a bivariate scatter plot of fluorescence versus forward scatter, by gate setting with normal LN cells. A minimum of 10,000 events from the viable transfected cell population per sample were analyzed and percentage of their fluorescence intensities were measured with respect to uninfected LN cells as control. Parasite inhibition was measured in terms of the decrease in expression of green fluorescence in the FP of vaccinated animals, compare to the lowest and highest levels of this parameter in the non-vaccinated control groups. PI% was calculated using Eq. (1) and IC95% values were also determined for each average of parasite inhibition of each group. This was calculated by comparing the intensity of fluorescence in vaccinated and non vaccinated groups using Eq. (1).

#### C-Cytokine assay

Prior to and 9 weeks post challenge, 3 mice form each group were sacrificed and LNs were existed, homogenized and washed. LN cells were plated in triplicates in 96-well microtiter plates in RPMI 5% (RPMI 1640 supplemented with 5% FCS, 1% L-glutamine, 1% HEPES, 0.1% 2ME, 0.1% gentamicine) as final count of 10^6^ per well and stimulated with 10 µg of the corresponding recombinant proteins (rCPA, rCPB, rCPB^-CTE^), SLA (soluble *Leishmania* antigen) or Concanvalin A (5 µg/ml, ConA), or culture medium (RPMI). Cell culture plates were incubated for 5 days at 37°C in 5% CO_2_ humidified atmosphere. Production of IFN-γ and IL-5 were measured in supernatant of stimulated LN cell culture by sandwich-based Elisa kits (R&D, Minneapolis, MN, USA) according to manufacturer's instruction. The lower detection limits of IFN- γ and IL-5 were 2 and 7 pg/ml, respectively. All tests were performed in triplicates.

#### D-Determination of antigen specific isotypes IgG1, IgG2a

Pooled sera were prepared from each group of mice before, and 9 weeks after challenge. Productions of IgG1 and IgG2a antigen specific antibodies were measured against SLA, rCPA, rCPB and rCPB^-CTE^. Briefly 96 well plates were coated with recombinant proteins or SLA antigen (10 µg/ml in PBS) and incubated at 4°C overnight. After 3 washes with washing buffer (PBS plus 0.05% tween20), plates were blocked with blocking buffer (1% BSA in PBS) for 2 hours at 37°C and then, 100 µl of the pooled sera, diluted 1∶50 in blocking buffer were added and incubated for 2 hours at 37°C. Biotinylated anti mouse IgG1 (1∶10000, Zymed, Burlington, ON, Canada) and IgG2a (1∶500, southern Biotech, Birmingham, USA) were added and incubated for 2 h at 37°C. Streptavidine-horseradish peroxidase (BRL, Gaithersburg, MD) was added (1∶1000) and incubated for 1 h at 37°C. Conjugates' binding were visualized with OPD (complete name) and the reaction was stopped by sulfuric acid (4N) and the absorbance was measured at 492 nm.

### Statistics

Statistics were performed using Graph-Pad Prism 5.0 for Windows (*Graphpad* Software Inc 2007, San Diego, Calif., USA). All the data analyzed with one way ANOVA (Multiple comparisons Tukey post test) when required, with the exception of size and zeta potential measurements, which were analyzed with a Student's t-test. A *p*-value of ≤0.05 was considered as significant difference between the groups. “n” represents number of mice per group or samples per assay.

## Results

### Physicochemical characteristics and *in vitro* evaluations of the vaccine formulation revealed the potential effectiveness of cationic SLN for pDNA delivery

cSLN were produced by using the modified microemulsion and high shear homogenization method, cetyl palmitate and cholesterol as matrix lipid, DOTAP as charge carrier and Tween 80 as surfactant. Obtained nanoparticles were approximately 257±23 nm in size and positively charged with a zeta potential of +52±8 mV in milli Q water and size distribution of 0.34±0.08. This suspension was stable for 30 days (*p*<0.05). The SLN-pcDNA stable complexes (S_p_a, S_p_b and S_p_b^-CTE^) were prepared by pDNA adsorption on the surface of cSLNs via direct complexation with pcDNA-*cpa*, pcDNA-*cpb* and pcDNA- *cpb^-CTE^*, respectively. These formulations were also characterized according to their size, zeta potential and poly dispersity index (PDI, Table 1). The results indicated that S_p_a, S_p_b and S_p_b^-CTE^ cationic formulations had an average size of 244±12, 250±15 and 237±12 nm, respectively. There was no significant difference in the size of different preparations (*p*>0.05). S_p_a, S_p_b and S_p_b^-CTE^ had a mono disperse formulation as the PDI value was about 0.2 for all of them. The observed zeta potential revealed all the formulations were cationic (*ζ* potential = 22 to 27 mV) that is suitable for interaction with the negatively charged cell surface and the cell entry.

**Table 1:** cSLN formulations and their characteristics.

Formulation	Size (nm)	Zeta potential (mV)	PDI
cSLN	257±23	+52±8	0.326
S_p_a	244±12	+27±3	0.254
S_p_b	250±15	+22±3	0.264
S_p_b^-CTE^	240±12	+25±7	0.283

Nanoparticles were formulated from cetyl palmitate, cholesterol and DOTAP hydrochloride. Results represent mean±SD of three independent SLN preparations.

The agarose gel electrophoresis analysis was used to test S_p_a, S_p_b and S_p_b^-CTE^ formulations for their ability to condense pDNA through electrostatic interactions after preparation (data not shown). Gel retardation assay for SLN-pDNAs confirmed complete complexation between pDNA and cSLN at a DOTAP:pDNA ratio of 6∶1. Cationic SLN were able to protect pDNA against *DNase* I digestion as previously reported [17]. S_p_a, S_p_b and S_p_b^-CTE^ formulations were stable at refrigerated temperature (4±1°C) over one month storage. Endotoxin concentration in the cSLN formulations was 0.215 EU/50 ug.In our previous *in vitro* studies on COS-7 cells, we demonstrated a very low degree of toxicity of both cSLN and cSLN–pDNA complexes. Furthermore, flow cytometry analysis confirmed SLN-pDNA complexes were able to promote transfection of COS-7 cells at least for 72 hrs after treatment of these cells without a significant reduction in cell viability and *in vitro* CPs expression capacity [17].

### Disease progression monitoring by five different techniques revealed the efficacy of S_p_a/b^-CTE^ cocktail DNA vaccination

The efficacy of cocktail pDNA vaccines containing pDNAs encoding CPA/CPB, CPA/CPB^-CTE^, and the same cocktail pDNA vaccines formulated with cSLN (S_p_a/b and S_p_a/b^-CTE^) were evaluated by their capability to induce protection against *Leishmania* infection in the BALB/c mice model. It is worth to mentin that, no apparent sign of local intolerance such as redness, swelling, bruising, pain observed at the site of injection 1 and 24 hr after vaccine administration. Parasite inhibition in different groups was assessed on both FP and LN of animals. For this purpose, dynamic measurement with a metric caliper, parasite load determination by flowcytometry and imaging techniques were done on the infected mice FP. In parallel, the parasite load was assessed in the LN via microtitration and flowcytometry methods.

**Figure 1:**
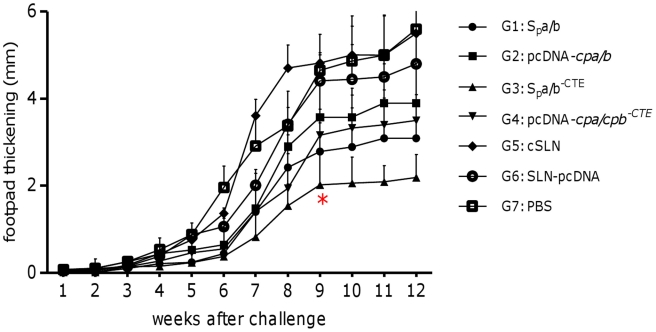
Assessement of footpad swelling in vaccinated mice. Schematic presentation of the mean footpad measurements via caliper based method in mm (left axis) with standard error over the course of the *Leishmania* infection following pcDNA-*cps* immunization. BALB/c mice, thirteen animals in each group, were immunized subcutaneously with cSLN-pcDNA-*cpa/cpb* (G 1); pcDNA-*cpa/cpb* (G 2); cSLN-pcDNA-*cpa/cpb^-CTE^* (G 3), pcDNA-*cpa/cpb^-CTE^* (G 4); cSLN (G 5), cSLN-pcDNA (G 6) and PBS (G 7). Mice were immunized again 3 weeks later with the same schedule. Three weeks after the booster immunization, mice were challenged in the left footpad with *L. major* 5days stationary phase promastigotes (3×10^6^ cells/mouse). Weekly measurements of footpad thickness represent the mean score ± SD in each group (*n* = 10). From the 9^th^ week, a statistically significant difference (*p<0.05) was seen between G3 and the control groups. This difference continued up to the end of study course. (“G”represents “group” in all the graphs).

As shown in Figure 1, FP swelling of seven different groups of mice n = 10)was measured after a challenge inoculation with EGFP-transfected stationary phase promastigotes of *L*. *major*. All the mice which have received the empty cSLNs (G5), pDNA vector without any insert formulated with cSLN (G6) or PBS (G7); showed significant lesions by 7 weeks post-challenge. There was a significant (*p*<0.05) difference between animals received formulation S_p_a/b and S_p_a/b^-CTE^ (G1 and 3) and animals immunized with non-formulated cocktail vaccines (G2 and 4) at 9^th^ week post challenge. The latter groups did not show any significant difference with the control groups. There was also a significant (*p*<0.05 difference between group 1 and 3, confirming that the higher level of protection observed in group 3. However, this difference between the cocktail vaccine compositions was not significant in group 2 and 4 and this difference could not be simply described due to the presence of CTE in the texture of the S_p_a/b cocktail vaccine. It seems that utilizing cSLNs for the formulation of pDNAs could potentiate the manifestation of this difference between the vaccine compositions. Vaccination with cSLN-pDNA encoding *cpa*, *cpb* (G 1) or cSLN-pDNA encoding *cpa*, *cpb^-CTE^* (G3) delayed FP swelling when compared to immunization with cSLN and PBS (G5 and 7), at this time point. For a certain time, immunization with the pcDNA-*cpa/b* and pcDNA-*cpa/b^-CTE^* cocktail vaccines had a significant effect in delaying FP swelling. However, this effect was not long lasting and 9 weeks after challenge, there was a significant difference (*p*<0.05) in protective effect between groups 3 and all of the other groups including group 1. Furthermore, the mean FP lesion size in group 1, was still significantly (*p*<0.05) lower than that in the control groups. Totally, vaccination with a cocktail of *cpa*, *cpb^-CTE^* formulated with cSLNs (G3) resulted in control of the infection progress compare to the control groups as all of the animals in this group developed significantly smaller FP lesions (2.02±0.7 mm) at 9^th^ week after challenge.

**Figure 2:**
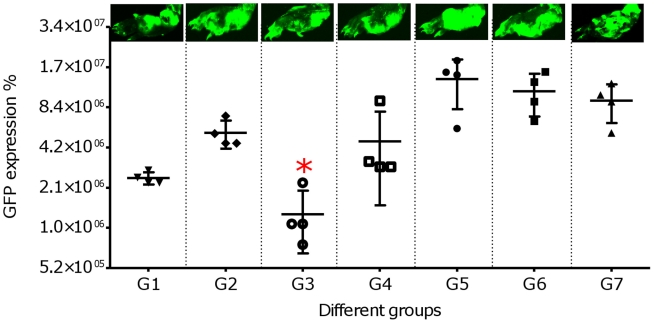
Fluorescence imaging of the footpads. Fluorescence imaging was performed at 9^th^ week after challenge Four mice from each group were selected randomly for this experiment, but one representative mouse of each group was chosen for the photographs, taken by fluorescence imaging system. Footpads from each group were averaged for each data point to demonstrate sum green pixel count in the fluorescence image at this time point. The mean of the sum green pixel count with standard error from footpad images plotted for each group. The vaccinated mice in group 3 showed significantly (*p<0.05, n = 4) lower footpad size and sum green pixel count from the other groups.

To assess the reliability of this conventional and time consuming experiment at 9^th^ week post challenge, we also detected EGFP-expressing *L. major* in the FPs of mice using fluorescence imaging system that is supposed to give a precise two-dimensional image from the extent of infection, independent of the inflammatory responses at the FP injection site. This experiment enabled us to clearly detect the parasite load in the infected FPs (Fig. 2). As shown in this Figure, the GFP fluorescence in the control group of animals was not localized to the site of the inoculation and the parasites spread to the whole FP. We also observed that the increasing thickness of the infected FPs was not correlated with the intensity enhancement of the detected GFPs in the tested animals (Fig. 2). The sum green intensity (pixel) from the imaging studies were higher in infected FP of the control groups. Furthermore, only group 3 of the tested animals revealed significantly (*p*<0.05, n = 4) lower GFP intensity compare to the other groups (Fig. 2).

**Figure 3:**
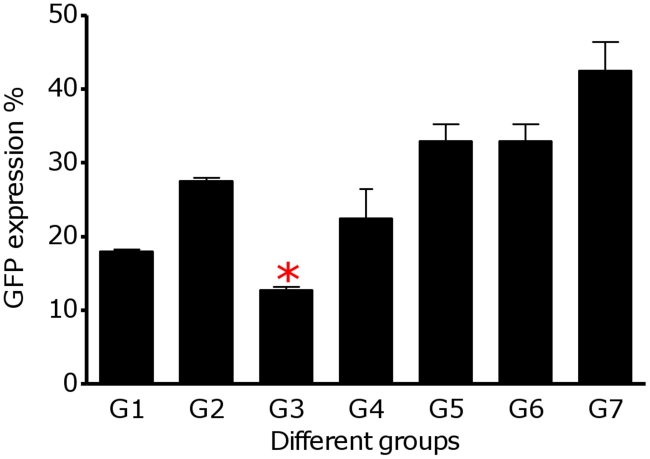
Flowcytometry analysis of the footpads. The bars represent the average percentage of GFP positive footpad cells ± SD of three mice per each group GFP fluorescence expression was significantly lower in the footpads of group 3 (*p<0.05).

EGFP expression in *L. major* amastigotes resident in the FPs was also monitored by flow cytometry technique. High expression levels of EGFP were observed in PBS treated group (G 7, Fig. 3). Fluorescence activated cell sorting (FACS) analysis indicated a clear quantitative separation between parasite transfected and normal cells. The frequency of EGFP positive cells of the FPs determined by FACS analysis using the appropriate gating are shown in Fig. 3. Group 3 of the vaccinated animals had the lowest infection rate (12.77%±0.62) compare to the other groups. The percentages of GFP-expressing parasites were significantly lower in the FP cells of the group 1 and 3 of the vaccinated animals.

**Figure 4:**
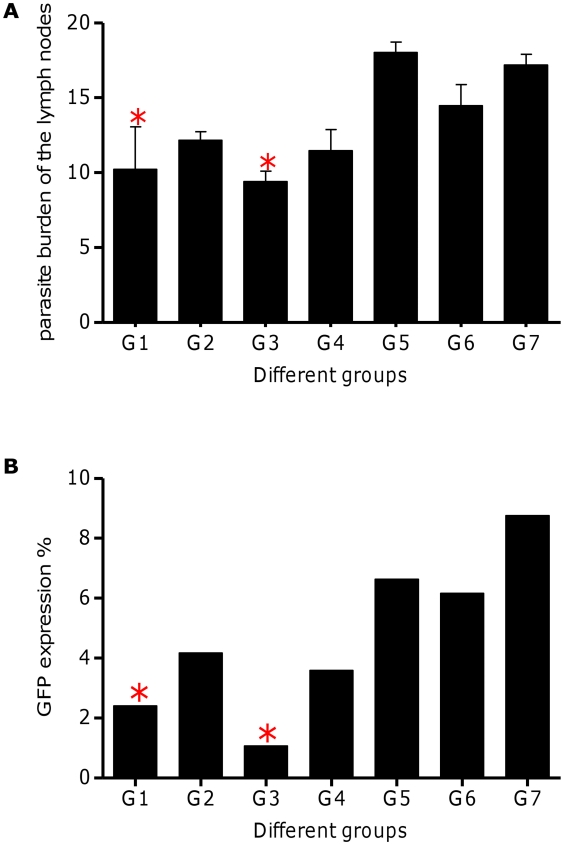
*L. major* burden of popliteal lymph nodes. (A) microtitration analysis: a limiting dilution analysis was performed 9 weeks after infection on the cells isolated from popliteal LN of 3 individual mice from each group and cultured in triplicate in Schneider's medium for 15 days at 26°C in serial 10-fold dilution. The wells were assessed microscopically for *L. major* growth, and the number of viable parasites was determined from the well with the highest dilution. The bar represents the average score±SD of three mice per group. Results are representative of at least two independent experiments and revealed a significant (*p<0.05) decrease in G1, G3 of the vaccinated mice compared to the control groups. (B) flowcytometry analysis. 9 weeks post-infection, intracellular fluoresence of the lymph node cells (pool of lymph nodes of three mice from each group) was quantified by Partec PASIII flow cytometer. “*” reveals significantly decrease in group 1 and 3 compared to unvaccinated control groups (*p*<0.05).

The infection progression was also followed by determining the total parasite load in the LNs of challenged mice at 9 weeks post-infection by the microtitration method (Fig. 4A) and flowcytometry (Fig. 4B). According to microtitration method and compare to control groups, parasite load in the LNs of vaccinated groups with S_p_a/b^-CTE^ (G3) decreased significantly (*p*<0.05, n = 3) (Fig. 4A). The expression of EGFP in the LNs was readily evident from the intense green fluorescence of the parasites (Fig. 4B). The parasites reside inside the LN cells were quantified by monitoring EGFP expression via flowcytometry analysis. As shown in Fig. 4B, Group 1 and 3 both expressed significantly (*p*<0.05, n = 3) lower percent of EGFP that is correlated with the least amastigote parasite existence in the lymphatic cells. On the other hand, parasite burden in the group 3 was significantly (*p*<0.05) lower than group 1.

It is noteworthy that, the rate of infection in all groups was in concordance with the delay in the appearance of lesions, the thickening peaked at 9 weeks post-infection and the parasite load in the LNs and FPs determined by microtitration, flow cytometry and imaging methods. Mice vaccinated with formulated cocktail plasmids i.e. S_p_a/b and S_p_a/b^-CTE^ (G1 and G3) showed a significant decrease in the FP tissues parasite load as well as LN, compared to the control groups. The ability of the vaccines to inhibit the infection progress and parasite replication [parasite inhibition (PI) %] was predicted according to the assessment of the FPs and LNs parasite load via imaging and flow cytometric method in terms of the decrease in intensity of green fluorescence observed in vaccinated animals, as well as parasite burden of the FPs and LNs by flowctometry and microtitration method. As shown in Table 2, the given rates were calculated by comparing the precentage of fluorescence intensity or parasite burden in FPs and LNs of vaccinated mice to the lowest and highest intensity of fluorescence in the non vaccinated groups. Average of PI% rates were more expressive, when FP imaging and LN flow cytometric techniques were used. The average PI% in G3 was significantly higher than other vaccinated groups when determined by different methods, executed on FPs and LNs. The mean average of parasite reduction in this group was 87.11% (IC95%, 85.9–88.33) by FP imaging, 65.64% (IC95%, 61.29–69.98) by FP flow cytometry, 41.43% (IC95%, 35.05–47.81) by LN microtitration and 86.77% (IC95%, 87.78–85.75) by LN flow cytometry (Table 2). The profile of the alteration in the PI% results in all of the vaccinated groups were correlated with the results of the LN parasite burden via microtitration assay and footpad swelling. Neither imaging nor flow cytometry assays could discriminate between the parasite inhibition results of G1 and G3 of the vaccinated animals, when manipulating the animals' footpads (Table 2).

**Table 2:** Average parasite inhibition percent (PI%) by different methods.

Determination Method	Average Parasite Inhibition (%)				Effective formulation(s)	Discrepancy between Effective formulations (*p<0.05)			
	G1	G2	G3	G4		G1vsG2	G1vsG3	G2vsG4	G3vsG4
FP Caliper based measurement (Fig. 1)	Not applicable				S_p_a/bS_p_a/b^-CTE^	*	*	NS	*
FP imaging(Fig. 2)	77.72±5.78	47.59±6.99	87.11±1.72	54.71±6.04	S_p_a/bS_p_a/b^-CTE^	*	NS	NS	*
FP flowcytometry(Fig. 3)	51.6±8.6	25.35±13.3	65.64±6.15	39.42±10.84	S_p_a/b^-CTE^	*	NS	NS	*
LN microtitration(Fig. 4A)	36.28±9.8	24.17±11.68	41.43±9.02	28.51±11.02	S_p_a/b^-CTE^	*	*	NS	*
LN flowcytometry(Fig. 4B)	66.82±8.2	41.35±14.4	86.77±1.4	50.37±12.23	S_p_a/bS_p_a/b^-CTE^	*	*	NS	*

Average PI% measured in terms of the parasitic load decrease in the footpad (FP) and/or lymph node (LN) of vaccinated animals compared to the control groups via different methods. (“NS” represent as “not significant” and “*” represents significant difference between the mentioned groups).

### Cocktail DNA vaccination with S_p_a/b^-CTE^ formulation encoding CPA, CPB^-CTE^ enhanced the IFN-γ production

**Figure 5:**
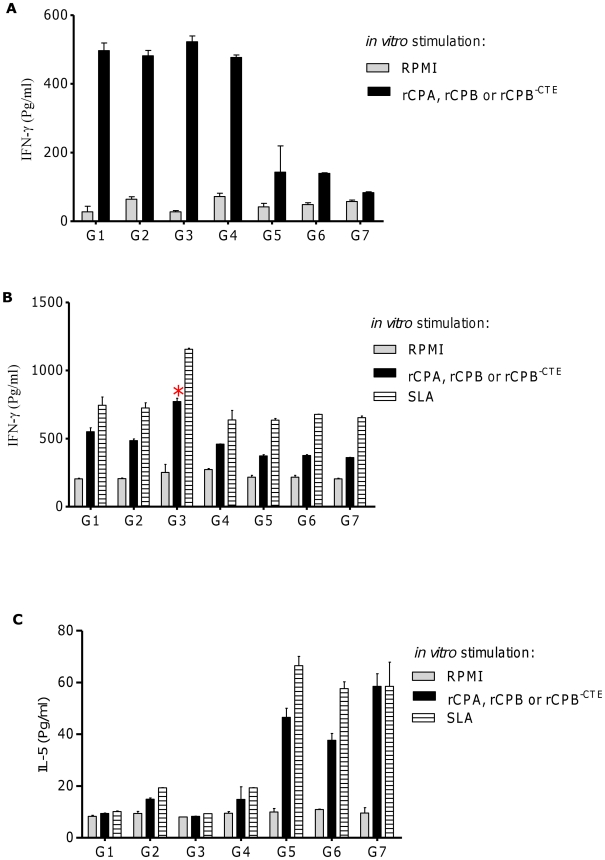
Cytokine production by lymph node (LN) cells from *L. major*-infected mice. Single-cell suspensions were prepared from the LN of three mice in the designated groups, before (n = 13) and 9 weeks after infection (n = 10). Cells cultured in triplicate for 5 days in the presence of recombinant antigens (10 µg/ml), soluble *Leishmania* antigen (SLA, 4 µg/well), Con A (as positive control) and RPMI (as negative control). Culture supernatants were assayed for levels of IFN-γ, before challenge (A), after challenge (B) and IL-5 (C) production by ELISA. There were no difference in Con A-induced cytokine production, among the groups. Each bar represents the mean ± SD for three mice per group (n = 3). Results are representative of two independent experiments, each performed in triplicate.

In order to compare the induced immune responses by different DNA cocktail vaccination strategies and explore the protective effects against *L. major* challenge in BALB/c mice, IFN- γ and IL-5 production were assessed after *in vitro* stimulation of LN cells with both *Leishmania* soluble antigen (SLA) and recombinant CPs, pre and post challenge. According to IFN-γ production, pooled cells from three mice of groups 1, 2, 3 produced significantly (*p*<0.05) higher levels compared to control groups before infection in response to the rCPs (Fig 5A). Although group 3 produced higher amounts of IFN-γ (522.98±11.99 pg/ml), but was not significantly higher than the other vaccinated groups. Low IFN-γ production was detected in supernatants of LN cells of all three control groups in response to rCPs, before challenge. At 9^th^ weeks post challenge, the rprotein specific IFN-γ production level increased only in group 1 and 3 of the animal vaccinated with S_p_a/b and S_p_a/b^-CTE^ formulations, respectively. Although, the difference in IFN-γ production level was only significant (*p*<0.05), in group 3 (773.29±16.78 pg/ml) of animals compared to control groups (Fig. 5B). No IL-5 was detectable in the supernatant of cells from all groups after stimulation with rCPs before challenge (data not shown). In contrast, significant levels of IL-5 were detected in the supernatant of cells from group 5, 6 and 7, in response to rCPs SLA at 9^th^ week after infection, compared to all the vaccinated groups of animals (G1, 2, 3 and 4, Fig. 5C). There were no significatnt difference in Con A-induced cytokine production, among the tested groups.

**Figure 6:**
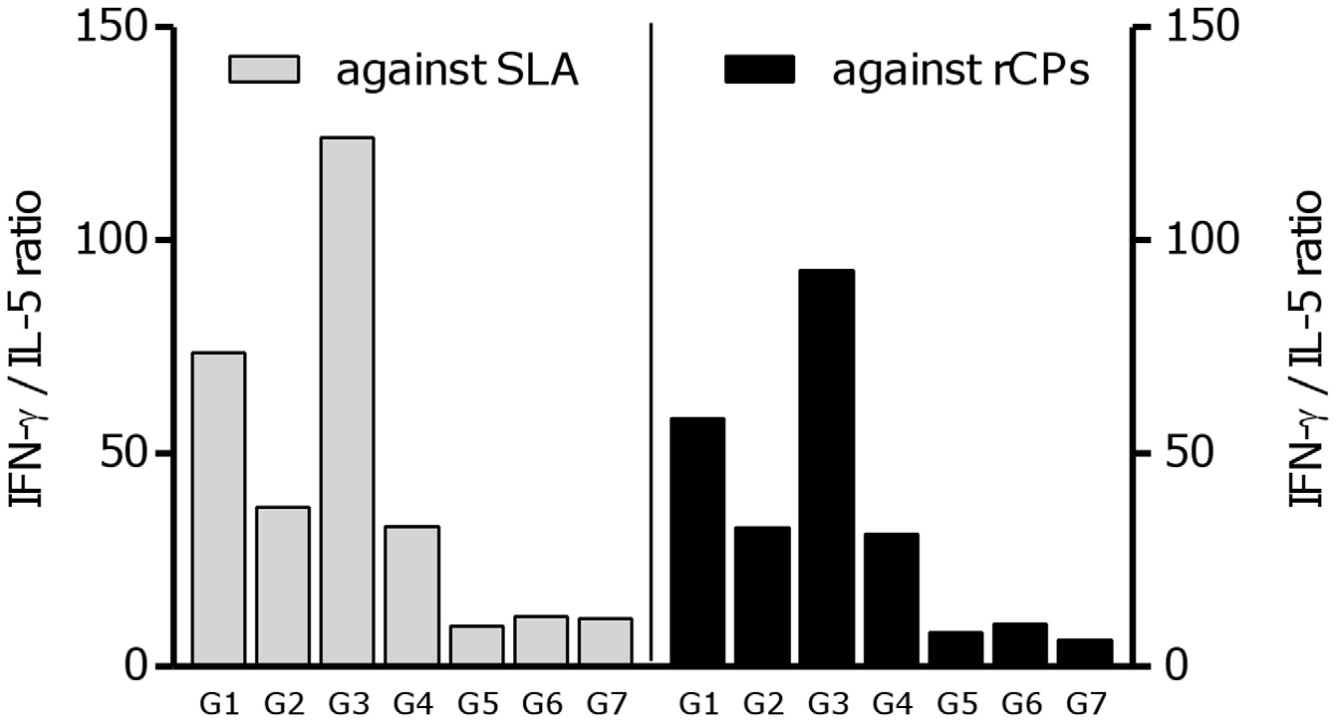
The ratio of IFN-γ/IL-5 production. The ratio presentation at week 9^th^ after challenge in the lymph node cells stimulated by SLA and respective rCPs. This ratio was significantly (*p<0.05) higher in the animals immunized with S_p_a/b^-CTE^ (G3).

Further analysis of the induced cytokines profile by means of IFN-γ/IL-5 ratio revealed that only formulated cocktail DNA vaccines (S_p_a/b and S_p_a/b^-CTE^) could clearly induced strong Th1 immune responses (Fig. 6). As shown in this Figure, this ratio was significantly (*p*<0.05) higher in group 3 of vaccinated mice compared to the other groups suggesting a higher level of protective immunity.

### CP-specific production of IgG2a correlated with protective effect of cocktail DNA vaccination with S_p_a/b^-CTE^

**Figure 7:**
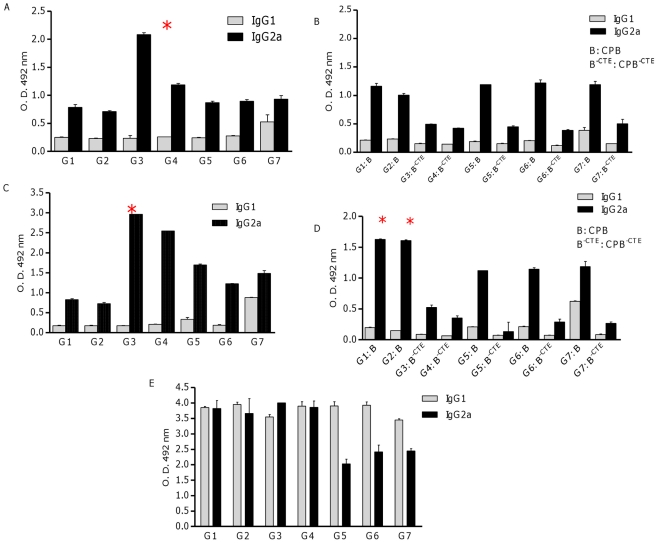
Antigen-specific IgG1 and IgG2a responses. Before and at week 9^th^ after challenge, blood samples were collected and pooled sera (diluted 1∶50) per each group was prepared. Antibody responses stimulated by rCPA (A, C) and rCPB or rCPB^-CTE^ (B, D) and SLA (E) showed the profile of CP-specific antibodies, induced before (n = 13) and after (n = 10) challenge. The data represent means±SD. Results are representative of two independent experiments, each performed in triplicate.

**Figure 8:**
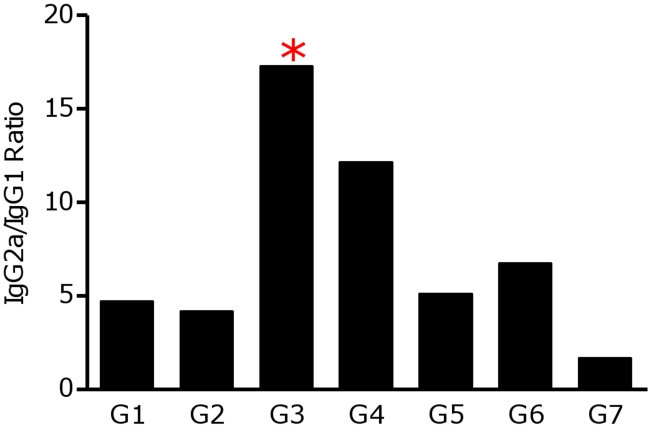
The ratio of IgG2a/IgG2a production. The ratio presentation at 9^th^ week after challenge in the different groups determined against rCPA. This ratio was significantly (*p<0.05) higher in the animals immunized with S_p_a/b^-CTE^ (G3).

To compare IgG isotypes in protected and non protected vaccinated groups, sera were collected before and 9 weeks after challenge and assessed for IgG1 and IgG2a. To determine the antibody specificity, all sera of vaccinated and control mice were pooled and assayed by ELISA method before (Fig. 7A, B and C) and after (Fig. 7D, E and F) challenge with *L. major* promastigotes. As it is shown in these Figures, the group of mice that developed an effective protective response (e.g., group 3) had substantially higher levels of CPA-specific IgG2a antibody compared to unprotected mice, both before (n = 13) and after (n = 10) challenge (Fig. 7A, C). This is consistent with the results obtained above that SLN formulated cocktail DNA vaccine encoding *cpa* and *cpb* ^-*CTE*^ (S_p_a/b^-CTE^) preferentially induced a Th1 response. According to the Fig. 7D, higher levels of CPB-specific IgG2a was produced in G1 and 2 of the vaccinated mice. This might be described by the immunogenic nature of CTE fragment. The productions of IgG1 in the control groups were significantly higher than IgG2a after challenge, when stimulated by SLA (Fig. 7E).The ratio of IgG2a/IgG1 in sera of mice immunized with S_p_a/b^-CTE^ formulation was higher than the other groups when titrated against rCPA (Fig. 8). This ratio was also higher in sera of mice immunized with the pcDNA-*cpa/b^CTE^* formulation than all the other vaccinated groups.

Challenged with GFP expressing *L. major* promastigotes induced high level of IgG1 antibody titers against SLA in all groups (Fig. 7E). The sera of mice immunized with S_p_a/b^-CTE^ formulation showed higher levels of specific IgG2a antibody compared to the IgG1, when titrated against SLA and higher IgG2a/IgG1 ratio was only observed in this group of vaccinated animals.

## Discussion

Although vaccination in the endemic populations is the most cost-effective tool to diminish the burden of *Leishmaniasis*, an effective vaccine to control this disease is not commercially available yet [2]. It is unlikely that an effective anti-*Leishmania* vaccine based on the use of a single antigen will be achieved. This might be due to the complex and biphasic life cycle of *Leishmania* parasite. Therefore, a rational approach toward developing an effective cocktail vaccine should be the use of extracellular and intracellular parasite antigens resulting in a valuable cumulative immune response [3], [14]. In this regard, ease of combining different pDNA vaccine candidates has made genetic vaccination an attractive platform for vaccination strategies [3], .

However in most cases, even multivalent or cocktail DNA vaccines have failed to achieve the required level of protection possibly due to the lack of an appropriate delivery system and/or adjuvant [3], [16]. Therefore, there is still an urgent need for development of new, safe and improved adjuvant and/or delivery systems to enhance the immunogenicity of the available vaccine candidates. There are versatile conflicting reports about DNA vaccination effectiveness against leishmaniasis. Most of DNA vaccine candidates have been tested as single vaccine regimens, but there are also some reports about using combination of genes [1], [3]. Up to date despite of the heterogeneity of vaccination protocols, mean average of parasite load reduction was determined to be 59.24% (IC95%, 47.75–70.73) [3]. This might be due to the reason that adjuvants and delivery systems were rarely added to the formulations containing candidates of the mentioned third-generation vaccines [3]. Approved adjuvants for human vaccines are poor inducers of antigen-specific Th1 responses that are necessary for an intra cellular parasite like *Leishmania* [21]. Therefore, several strategies, including live vectors; saponins; Freund's and montanide ISA 720 water-in-oil emulsions; oil-in-water emulsions (MF59); dendritic cells (DC) and liposomes have been utilized in different studies and trials to deliver antigens and redirect the immune responses towards desired Th1 pathway [22]. However, most of these failed to provide both long-term immunity and safety for human vaccines.

Therefore, it is still crucial to develop a potentiated delivery system with Th1 stimulating, safe and cost-effective properties for such a promising vaccination technology. Nanoscale vehicles are able to boost the quality and magnitude of an immune response in a predictable, designable trend that can be applied for wide-spread use of genetic vaccination, for developing vaccines for diseases such as cutaneous leishmaniasis, which is currently managed only through relatively ineffectual therapeutic regimens. Nanoparticles as vaccine delivery systems, promote bioactive vaccine candidate protection against extracellular degradation and modulate cellular and humoral immune responses via targeting antigens to APCs such as DCs and therefore would be potentially useful as effective vaccine adjuvants [15]. Cationic lipid-based systems could be formulated as emulsions, liposomes or cationic solid lipid nanoparticles (cSLN). These Lipid-based delivery systems are able to protect the nucleic acid payload and significantly reduce its degradation and extend its activity, improve the pDNA pharmacokinetic characteristics and thus induce more potent immune responses due to a depot effect in which persistence of pDNA at the site of delivery allows uptake by local immune cells, enhance intracellular uptake and delivery to target APCs [15]. Amongst these, SLNs have offered a number of technicopractical advantages including proper storage stability, easy production procedure, steam sterilization and lyophilization possibility and acceptable safety profile [7], [8]. In regards to vaccination studies, significant enhancement was reported when using cSLN-pDNA formulation for cholera toxin and lipid A delivery so that well tolerated particles with sizes greater than 100 nm exhibited higher adjuvant activity enhancing both T-helper types of immune responses compared to the FIA [8]. Hence, in this study we considered SLN for formulating *cpa* and cp*b* or *cpb^-CTE^* genes. In our previous study, these genes as DNA prime boost vaccination regimen have induced partial protective responses in susceptible BALB/c mice [14]. Heterologous prim-boost immunization approaches have complex logistics and high costs associated with manufacturing a second vaccine platform. Therefore, utilizing a suitable delivery system might be an important strategy to eliminate the need for boosting with the recombinant proteins and enhance third generation vaccine's potency by preventing rapid elimination of the administered pDNA from the circulation. In this regard, cSLN formulations were prepared and characterized regarding their size, zeta potential, nuclease protection, *in vitro* transfection efficiency, and cell viability, in our earlier report [17].

To assess the cSLN utility prospect as a leishmanial vaccine delivery system, we exploited GFP-transfected *Leishmania* for generating experimental cutaneous leishmaniasis in BALB/c mice. This model was reported as a novel dynamic immunopathogenic tool allowing visualization and correlation of fluorescence intensity with parasite burden [23], [24]. Some concerns might be raised according to an anti-GFP immune response which could be induced against expressed GFP by the parasites. Several studies indicated that no immune responses have been detected in animals immunized with recombinant EGFP. In other words, recombinant EGFP is not able to stimulate APCs, nor do it induce a significant T-cell response or anti-EGFP antibody production [25]. In our investigations for precise judgment about the feasibility of this delivery system, we looked for a more rapid, sensitive and easily reproducible method to predict average parasite inhibition (PI) in the FP and LN of the vaccinated animals. Therefore, different techniques were used in this regards and the outcomes were correlated to the conventional standard caliper-based method and microtitration parasite burden as well as cytokine and antibody responses to choose the most sensitive, precise and less time-consuming technique for following *Leishmania* infection, in mice model. As illustrated in Table 2, to evaluate the protection rate; FP swelling (Fig. 1), imaging (Fig. 2) and flowcytometric analysis on the FP (Fig. 3) and LN (Fig. 4B) were assessed in immunized mice and the results were compared to each other and the control groups. The LN parasite burden was also determined by microtitration conventional method (Fig. 4A), at the same time point. The results demonstrated that the size of lesion in mice immunized with S_p_a/b and S_p_a/b^-CTE^ at week 9 post challenge were significantly (*p<*0.05) smaller compared to control groups. Interestingly, the mice immunized with *cpa/cpb* and *cpa/cpb^-CTE^* had also revealed significant difference in FP swelling compared with the group of mice immunized with the formulated cocktail of pDNA*-cps* (*p*<0.05). However, there was no significant difference between the groups of mice received non-formulated cocktail vaccines. The same results were obtained through LN analysis by microtitration and flowcytometry. The mentioned methods disclosed the significant PI discrepancy between S_p_a/b and S_p_a/b^-CTE^ formulations, while FP analysis couldn't discriminate the effective vaccination strategy between these formulations. However, although FP caliper and LN microtitration based procedures were accurate, precise and capable of differentiation between effective formulations in terms of tissue prasitism, they were time-consuming, rather difficult and not absolutely reproducible as the risk of operator errors are more probable in these experiments. Therefore according to the presented data, it seems that parasite inhibition can be directly estimated by flowcytometry analysis performed on LN cells; as a more rapid, sensitive, and easily reproducible method for screening anti *Leishmania* vaccine candidates and delivery systems.

The number of viable *L. major* was quantitated in the FPs and LNs of vaccinated groups of mice after challenge and compared to the control groups, and used as an indication of the protection rate or average parasite inhibition percent (PI%). The significantly (*p*<0.05) higher mean PI% was seen in group 3 (73.36±15.94%) which was immunized with S_p_a/b^-CTE^ formulation. This result further revealed the adjuvant effect of the cSLN for potentiating the immunogenicity of this genetic vaccination strategy (Fig. 1, 2, 3, 4A and B) and (Table 2). It seems that a/b^-CTE^ cocktail vaccine induced lower levels of the protection and needs a suitable delivery system to maintain and enhance its immunoprotective activity (44.38±12.59%). This obtained protection rate is in accordance with the percents of reduction of parasite load, reported to obtain with DNA vaccination, without a booster injection (59.24%) [3]. However, despite using cSLN, S_p_a/b formulation couldn't provoke the same PI%. as did S_p_a/b^-CTE^ (58.31±17.61%). This difference could be better described by the antigenic nature of the pDNAs used in this experiment and further confirms our previous results that CTE is highly immunogenic but not protective and more favorable to direct the immune system responces towards Th2 type. Based on the presented data, cSLN formulation conferred immunoprotecting activity to a/b^-CTE^ genes which were non-immunoprotecting in their free form, and possibly enhance the immunostimulatory activity of these genes, by effectively inducing TLR-9 mobilization in the endosomal compartment. According to the data presented in Table 2, despite utilizing highly sensitive methods to determine parasite burden, there was no significant difference between *cpa/cpb* and *cpa/cpb^-CTE^* cocktail DNA vaccines (G2 vs G4; Table 2). On the other hand, the discrepancy between these cocktails was significant when they were formulated with cSLN and the parasite burden was determined by LN manipulation (G1 vs G3, Table 2). This data further supports the importance of the lymph nodes as one of the most relevant tissues involved in the parasite-host interface during the stages of *L. major* infection as the cellular and humoral immune responses in the LN are able to better describe the major immunological changes due to parasite persistence during infection. Therefore, LN may reflect the profile of the host's immune response and the parasite burden intensity throughout *L. major* infection via both conventional (microtitration) and novel (flow cytometry) techniques. PI% were singnificantly different between the groups received formulated and non formulated cocktail vaccines (G1 *vs* G2 and G3 *vs* G4). These results emphasis induction of the immune responses by using delivery systems in such an extent that even the effect CTE deletion could be detectable in the disease progression.

To further evaluate the precision of the obtained data, the cytokines produced by antigen-specific T-cells, were evaluated to determine the profile of an elicited antibody response. IL-5 is associated with high levels of IgG1, whereas production of IgG2a is dependent on IFN-γ. Our results demonstrated that the immune response elicited by S_p_a/b and S_p_a/b^-CTE^ formulations was dominantly Th1 response denoted by the higher ratio of IFN-γ/IL-5 secretion after stimulation with SLA and rCPs. However this ratio was significantly higher in S_p_a/b^-CTE^ vaccinated animals. This ratio is approximately 3-fold higher when SLN has been used as a delivery system. T-cell immunogenicity of CPs had been shown in previous studies, where immunizations with *L. mexicana* recombinant CP resulted in the development of a potentially protective Th1 cell line, and that recombinant CPB from *L. major* efficiently induced CD8+ T-cells [9], [26]. Therefore, SLN proceeded as a Th1 stimulator adjuvant and increased the cocktail vaccine efficiency in elicitation of protective responses.

IgG1 and IgG2a antibody titers were also used as an indicator of Th2 and Th1 immune responses, respectively. The significantly (*p<*0.05) highest IgG2a was seen in the sera of group 3 of mice immunized with S_p_a/b^-CTE^ before challenge against recombinant CPA compared to other groups. This might be an indication that CTE-domain deletion in S_p_a/b^-CTE^ formulation redirected the immune responses toward increasing IgG2a production. However, using cSLN formulation for this cocktail vaccine consequently induced a more potent antibody response compared with free pDNAs (Fig. 7A, C and G3, G4). At week 9^th^ after challenge, the IgG2a/IgG1 ratio in mice vaccinated with S_p_a/b^-CTE^ formulation (G3) was correlated with a Th1 response and further confirmed that could induce a potent Th1 type of immune response and protection against leishmaniasis, at least in murine model. Only G3 showed significantly (*p*<0.05) highest ratio of IgG2a/IgG1, revealing the induced protection in this group confirmed by a significant smaller FP swelling (*p*<0.05), lower LN parasite load (*p*<0.05), highest IFN-γ/IL-5 ratio (*p*<0.05) and maximum average of parasitic inhibition percent by flowcytometry and imaging methods.

Despite, using cSLN as a delivery system, the immune responses to the selected antigens (*cpb* and *cpb^-CTE^*) of the cocktail vaccine were differed. Generally, the titres of specific antibodies raised by DNA vaccination are lower than those obtained after vaccination with a recombinant protein. As it is shown in this study antibodies are induced to a very low extent against CPB^-CTE^, especially in comparison to antibody levels against CPB (Fig. 7B, D). This is definitely attributed to the presence of immunogenic CTE-domain in the vaccine formulations administered to G1 and G2 that affect the character and potency of the responses against defined antigens in the mentioned cocktail vaccines. As a part of our experiments, we have performed an ELISA test in which pooled sera of G1 and G2 were tested against CPB^-CTE^ and sera of G3, G4 tested against recombinant CPB. As a result, G1 and G2 revealed reduced antibody responses while G3, G4 did not show any difference in antibody responses (data not shown). Thus, to avoid presenting exaggerated responses in the benefit for Spa/b^-CTE^ formulation, the presented data show the results of the experiment in which each group plated against its own set of antigens. Therefore, CTE-domain deletion is shown to be an appropriate approach to design a protective vaccine candidate against *L. major* as well as *L. infantum* infectious challenge [13].

Above mentioned protective Th1 response demonstrated in G3 that was characterized by increased titres of IgG2a in sera and elevated IFN-γ production by LN cells both before and after challenge, was further supported by the report by Brewer J M *et al*. indicated that lipid vesicles with a mean diameter >225 nm preferentially induces Th1 responses in BALB/c mice [27]. On the other hand, pcDNA-*cps* also possesses several immunostimulatory CpG motifs within pcDNA3.1 vector backbone. These CpG motifs might also facilitate priming of CTL responses by activating DCs [28]. Nevertheless, this effect is often temporary because of the rapid degradation of DNA [5], [15] and consequently repeated administrations or much higher doses are required to achieve the desired effects. Moreover, the pDNA delivery to the intracellular compartments for recognition by TLR-9 is hardened [5], [29] without a delivery system, as shown for G2 and G4. Therefore, we can conclude that cSLN as a delivery systems improved the storage stability [17], transfection efficiency [17] and immunostimulatory effects of pDNA-*cps* (Table 2).

This was possibly the result of pDNAs protection from nuclease activity *in vivo*, as we have previously reported this potential *in vitro* [17] and facilitation of pDNAs delivery to the cytoplasm because of cSLN positive zeta potential and the presence of cholesterol domains in cSLN formulation that enhances transfection efficiency by facilitating membrane fusion [17], [30]. In addition, phagocytosis of cSLN-pDNAs by APCs as well as localization of them in the draining LNs occurs easily following SC administration due to the composition and physicochemical characterization of these nanoparticles. The presence of Tween 80 in the formulation enhances this phenomenon as recently Seeballuck and co-workers have also demonstrated that, this surfactant would increase lymphatic uptake by promoting chylomicron formation [31]. Since the draining LNs contain a greater number of cells that express TLR9, localization of pDNAs in the draining LNs possibly will be an important mechanism by which cSLN formulation has enhanced the immunological activity of these antigens. Another important aspect in this formulation could be the presence of DOTAP in this cSLN formulation that can also activate the dendritic cells through a common binding partner with LPS [32]. Therefore, this formulation not only act as a delivery system but also as an adjuvant for *Leishmania* vaccine by improving the uptake of loaded antigens and also stimulating immune cells in specific way.

In conclusion, this paper clearly demonstrates that cSLN is a promising and adaptable delivery system that can be modified rationally towards specific vaccine targets by varying composition. Simplicity, reproducibility and the scale up possibility of the manufacturing process together with the appropriate immunostimulatory effects of this formulation as a delivery system might be utilized to create a stronger protective vaccine in combination with *Leishmania CPs.* The current data, in murine model of *L. major* infection, showed promising role of cSLN as an adjuvant to enhance stronger immune response against *Leishmania* infection. The mean average of parasite load reduction for such a cocktail pDNA vaccination was determined to be 38% [1]. Here in this study, we report that the percent of parasite inhibition by a particulate cocktail DNA vaccination technology could be increased up to 73.36±15.95%, according to the precise methods for parasite burden determination in the different organs of the challenged animals via both conventional (i.e. microtitration, Fig. 4A) and more novel (i.e. flow cytometry, Fig. 3, 4B and imaging, Fig. 2) techniques. In the entire mentioned techniques parasite burden gave a discriminative view among control groups and S_p_b^-CTE^ vaccinated animals. Amongst the disscussed methods, direct LN flowcytometry was found to be the most rapid, sensitive, and easily reproducible method for screening vaccination strategies. These promising data warrant further investigations in this regard. Our future studies are being designed to expand cSLN passive targeting to an active targeting to increase the vaccination efficiency. Coating cSLN harbouring pDNA-cps with ligands (such as mannan) are our main future visions to increase the cellular immune responses.
